# Facile Synthesis and X-ray Attenuation Properties of Ultrasmall Platinum Nanoparticles Grafted with Three Types of Hydrophilic Polymers

**DOI:** 10.3390/nano13050806

**Published:** 2023-02-22

**Authors:** Abdullah Khamis Ali Al Saidi, Adibehalsadat Ghazanfari, Shuwen Liu, Tirusew Tegafaw, Mohammad Yaseen Ahmad, Dejun Zhao, Ying Liu, So Hyeon Yang, Dong Wook Hwang, Ji-ung Yang, Ji Ae Park, Jae Chang Jung, Sung-Wook Nam, Yongmin Chang, Gang Ho Lee

**Affiliations:** 1 Department of Chemistry, College of Natural Sciences, Kyungpook National University, Taegu 41566, Republic of Korea; 2 Department of Medical & Biological Engineering, Kyungpook National University, Taegu 41944, Republic of Korea; 3 Division of RI-Convergence Research, Korea Institute of Radiological & Medical Science, Seoul 01817, Republic of Korea; 4 Department of Biology, College of Natural Sciences, Kyungpook National University, Taegu 41566, Republic of Korea; 5 Department of Molecular Medicine, School of Medicine, Kyungpook National University, Taegu 41944, Republic of Korea

**Keywords:** ultrasmall Pt nanoparticle, hydrophilic and biocompatible polymer coating, high X-ray attenuation power, computed tomography contrast agent

## Abstract

Ultrasmall platinum nanoparticles (Pt-NPs) grafted with three types of hydrophilic and biocompatible polymers, i.e., poly(acrylic acid), poly(acrylic acid-co-maleic acid), and poly(methyl vinyl ether-alt-maleic acid) were synthesized using a one-pot polyol method. Their physicochemical and X-ray attenuation properties were characterized. All polymer-coated Pt-NPs had an average particle diameter (d_avg_) of 2.0 nm. Polymers grafted onto Pt-NP surfaces exhibited excellent colloidal stability (i.e., no precipitation after synthesis for >1.5 years) and low cellular toxicity. The X-ray attenuation power of the polymer-coated Pt-NPs in aqueous media was stronger than that of the commercial iodine contrast agent Ultravist at the same atomic concentration and considerably stronger at the same number density, confirming their potential as computed tomography contrast agents.

## 1. Introduction

Over the past decade, nanoparticles (NPs) have been extensively investigated owing to their excellent physicochemical properties and resulting potential biomedical applications [1,2,3]. Among them, platinum NPs (Pt-NPs) have demonstrated a range of biomedical applications owing to their diverse properties, including enzyme-like catalytic properties applicable to nanozymes [4,5,6,7], antioxidant effects that extend the lifespan of living objects [8,9], cancer therapeutic effects [10,11,12], plasmonic effects applicable to photothermal therapy and radiotherapy [13,14], antibacterial effects [15], photoacoustic imaging properties [16], and high X-ray attenuation power [17] applicable to X-ray computed tomography (CT) contrast agents. In addition, they have exhibited low toxicity [18,19,20].

Contrast agents are highly valuable materials that facilitate the diagnosis of disease via contrast enhancements [21,22]. Magnetic resonance imaging (MRI) and CT are the most popular imaging modalities for diagnosing diseases, as they provide high-resolution three-dimensional anatomical images of the body. The imaging resolution and sensitivity can be further enhanced with contrast agents. Notably, high-contrast images of blood vessels and soft tissues can be obtained with CT in the presence of CT contrast agents [23]. Various heavy metal-based NPs, such as gold NPs [23,24], bismuth sulfide NPs [25], tantalum oxide NPs [26,27,28], lanthanide oxide NPs [29,30,31], and Pt-NPs [16,32,33,34,35,36], have been investigated as CT contrast agents owing to their higher X-ray attenuation coefficients compared with those of iodine contrast agents [17].

Pt-NP contrast agents in particular have several additional advantages with respect to iodine contrast agents. Iodine contrast agents with three or six iodine atoms per molecule generally possess high osmolality [23,37], which can cause side effects. They exhibit rapid renal excretion owing to their low molecular mass, allowing for only short imaging times. Moreover, their disease-specific imaging via conjugation with antibodies or targeting ligands is poor. Conversely, Pt-NP contrast agents, owing to a higher X-ray attenuation coefficient of Pt [17] and a high density of Pt atoms per nanoparticle, can yield considerably higher contrasts compared with those of iodine contrast agents. In addition, they can provide a lower osmolality than iodine contrast agents owing to their lower number density at the same atomic concentration. They can provide much longer blood retention times than iodine contrast agents owing to their considerably higher nanoparticle mass, allowing longer imaging times after intravenous injection. They can also serve as drug delivery vehicles after conjugation with antibodies or disease-specific ligands on their surfaces, allowing disease-specific diagnosis and therapy. Yet, despite the above-mentioned advantages, studies on Pt-NP contrast agents, as well as other heavy metal-based NP contrast agents, remain limited.

NPs must be stable in the colloidal form in aqueous media and non-toxic for biomedical applications. Thus, they should be grafted with biocompatible and hydrophilic polymers. In this study, poly(acrylic acid) (PAA) [38,39,40], poly(acrylic acid-co-maleic acid) (PAAMA) [41,42,43], and poly(methyl vinyl ether-alt-maleic acid) (PMVEMA) [44,45] were chosen because they are biocompatible and possess abundant hydrophilic carboxylic groups for binding to Pt-NPs. Moreover, previously, they had been successfully grafted onto lanthanide oxide NPs, exhibiting both low toxicity and excellent colloidal stability, showing no precipitation for over one year after synthesis [39,41,45].

Herein, we used for the first time, a one-pot polyol method to synthesize ultrasmall Pt-NPs coated with PAA, PAAMA, and PMVEMA and characterized their physicochemical and X-ray attenuation properties. Compared to previously reported synthesis methods [16,32,33,34,35,36], the one-pot polyol method used in this study is considerably simpler. The results showed that the synthesized polymer-coated Pt-NPs were nearly monodispersed and ultrasmall in particle diameter, and thus smaller than those of the previously reported Pt-NPs [16,32,33,34,35,36]. In vitro cellular toxicity was assessed to ensure low toxicity of the polymer-coated Pt-NPs. Finally, X-ray attenuation power was estimated, and the results were compared with those of a commercial iodine CT contrast agent, Ultravist (Bayer, Leverkusen, Germany).

## 2. Materials and Methods

### 2.1. Chemicals

Platinum (IV) chloride (PtCl_4_, 96%), sodium hydroxide (NaOH, 99.99%), PAA (Mw = ~1800 amu), PAAMA (Mw = ~3000 amu), PMVEMA (Mn = ~80,000 amu), triethylene glycol (TEG) (99%), potassium bromide (KBr, ≥99% trace metals basis), Whatman filter paper (qualitative, 70 mm), and dialysis tube [molecular weight cut off (MWCO) = 2000 amu, benzoylated cellulose tubing, 32 mm] were purchased from Sigma-Aldrich (St. Louis, MO, USA) and used as received. Ethanol (99%, Duksan, Ansan, South Korea) was used for the initial washing of polymer-coated Pt-NPs. Triple-distilled water was used for the final washing of the polymer-coated Pt-NPs and preparation of NP colloidal solutions.

### 2.2. Synthesis

**Figure 1:**
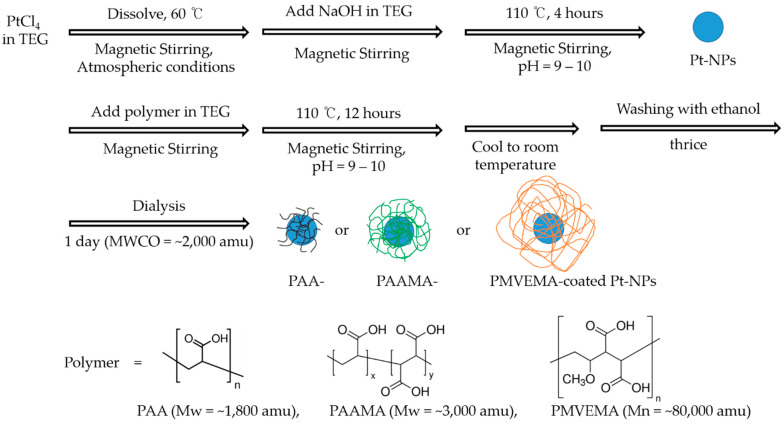
One-pot polyol synthesis of ultrasmall Pt-NPs coated with hydrophilic and biocompatible polymers: polymer = PAA or PAAMA or PMVEMA.

Ultrasmall Pt-NPs coated with hydrophilic and biocompatible polymers (i.e., PAA, PAAMA, and PMVEMA) were synthesized using a one-pot polyol method (Figure 1). Briefly, three different solutions were prepared, (i) a precursor solution prepared from 1 mmol of Pt precursor in 15 mL of TEG in a 100 mL three-necked round-bottom flask via magnetic stirring at 60 °C under atmospheric conditions (to maintain a constant temperature, the three-necked round-bottom flask was suspended in a silicone oil bath on a hot plate); (ii) an NaOH solution prepared from 9 mmol of NaOH in 20 mL of TEG in a 100 mL beaker via magnetic stirring at 60 °C under atmospheric conditions; and (iii) a polymer solution comprising 0.5 mmol of PAA (or 0.4 mmol of PAAMA or 4.5 × 10^−3^ mmol of PMVEMA) in 15 mL of TEG in a 100 mL beaker via magnetic stirring at room temperature under atmospheric conditions. The NaOH solution was slowly added to the Pt-precursor solution with constant magnetic stirring until the pH of the solution reached 9–10. Subsequently, the solution was slowly heated to 110 °C with magnetic stirring, and this temperature was maintained for 4 h. The polymer solution was slowly added to the Pt-precursor solution with magnetic stirring for 12 h, and the NaOH solution was added to maintain the solution pH between 9–10. The product solution was cooled to room temperature and then poured into a 1 L beaker, followed by the addition of 500 mL ethanol and magnetic stirring for 30 min. The diluted product solution was stored in a refrigerator (4 °C) until the polymer-coated Pt-NPs settled at the bottom of the beaker. The top transparent solution was decanted, and the remaining product solution was washed thrice with ethanol using the same process. To remove ethanol from the polymer-coated Pt-NPs, 400 mL of triple-distilled water was added to the product solution, which was subsequently reduced to approximately 50 mL via rotary evaporation. The product solution was further dialyzed using a dialysis tube for one day against 1 L of triple-distilled water with magnetic stirring to remove any remaining impurities; the triple-distilled water was replaced thrice during dialysis. One-half of the sample was dried to powder form for various characterizations, and the remaining half was dispersed in triple-distilled water to prepare an aqueous NP colloidal solution sample.

### 2.3. General Characterizations

The particle diameters of the polymer-coated ultrasmall Pt-NPs (polymer = PAA, PAAMA, and PMVEMA) were measured using a high-resolution transmission electron microscope (HRTEM) (Titan G2 ChemiSTEM CS Probe, FEI, Hillsboro, OR, USA) operated at an accelerating voltage of 200 kV. For measurements, one drop of diluted NP colloidal sample in triple-distilled water was placed onto a carbon film supported by a 200-mesh copper grid (PELCO no. 160, Ted Pella, Inc., Redding, CA, USA) placed on Whatman filter paper using a micropipette (2–20 mL, Eppendorf, Hamburg, Germany). The copper grid containing the sample was air-dried at room temperature before being loaded into the HRTEM. The scanning transmission electron microscopy (STEM) mode was used to obtain high angle annular dark field (HAADF)-STEM images. An energy-dispersive X-ray spectroscopy (EDS) instrument (Quantax Nano, Bruker, Berlin, Germany) installed inside the HRTEM was used to demonstrate the presence of Pt in the nanoparticle samples. 

Hydrodynamic diameters (a) and zeta potentials (ζ) of polymer-coated ultrasmall Pt-NPs in aqueous media were measured using diluted solution samples (0.1 mM [Pt]) and a particle size analyzer (Zetasizer Nano ZS, Malvern Panalytical, Malvern, UK).

The crystal structure of the powder samples before and after thermo-gravimetric analysis (TGA) was measured using a powder X-ray diffraction (XRD) spectrometer (X-PERT PRO MRD, Philips, Eindhoven, The Netherlands) with unfiltered CuKa (λ = 1.54184 Å) radiation. The scanning step and scan range in 2θ were 0.03° and 15–100°, respectively.

The grafting of the polymers onto ultrasmall Pt-NP surfaces was investigated by recording the Fourier transform-infrared (FT-IR) absorption spectra (Galaxy 7020A, Mattson Instruments, Inc., Madison, WI, USA). For measurements, the powder samples were dried on a hot plate at 40 °C for one week. Pellets of the dried powder samples were prepared in KBr. FT-IR absorption spectra were recorded in the range of 400–4000 cm^−1^.

The amount of polymer coating on Pt-NP surfaces was estimated by recording TGA curves using a TGA instrument (SDT-Q600, TA Instruments, New Castle, DE, USA) between room temperature and 700 °C under nitrogen flow. The amount of surface coating (wt. %) was estimated from the mass drop in TGA curves after subtracting the initial mass drop between room temperature and ~100 °C owing to water and air desorption.

The Pt concentration in NP colloidal solution samples was measured using an inductively coupled plasma-atomic emission spectrometer (Optima 7300DV, PerkinElmer, Waltham, MA, USA). The colloidal solution samples were pre-treated with acids to completely dissolve the Pt-NPs before measurements. Origin 8.5 (OriginLab, Northampton, MA, USA) was used for data analysis and plot.

### 2.4. In Vitro Cell Viability Assay

The in vitro cellular cytotoxicity of polymer-coated Pt-NPs on the normal human embryonic kidney (HEK293) and human liver tumor (HepG2) cell lines was assessed using a luminescent cell viability assay (CellTiter-Glo, Promega, Madison, WI, USA). The cells were seeded in a 24-well cell culture plate (5 × 10^4^ cell density, 500 μL cells/well) and subsequently incubated for 24 h (5% CO_2_, 37 °C). Four test NP colloidal samples (5, 10, 15, and 20 μM [Pt]) were prepared by diluting concentrated NP colloidal solution samples with sterile phosphate buffer saline solution. Approximately 2 μL of each test NP solution sample was added to the cultured cells, and the cells treated with the test NP solution samples were then incubated for 48 h. Adenosine triphosphate was quantified using a luminometer (Victor 3, PerkinElmer, Waltham, MA, USA), and cell viability was measured thrice and normalized to that of the control cells (i.e., untreated cells with NP samples).

### 2.5. X-ray Phantom Image Measurements

X-ray phantom images were acquired using a micro-CT scanner (Inveon, Simens Healthcare, Erlangen, Germany). Further, X-ray attenuation power was estimated in Hounsfield units (HU) with reference to that of water (0.0 HU) using the formula, HU = 1000 (μ_sample_ − μ_water_)/μ_water_, where μ is the linear attenuation coefficient of the materials estimated from the phantom images. The measurements were recorded under the following conditions: X-ray source voltages, 50 and 70 kV_p_ and imaging time per frame, 300 ms.

## 3. Results

### 3.1. Physical Characteristics of Polymer-Coated Pt-NPs

**Figure 2:**
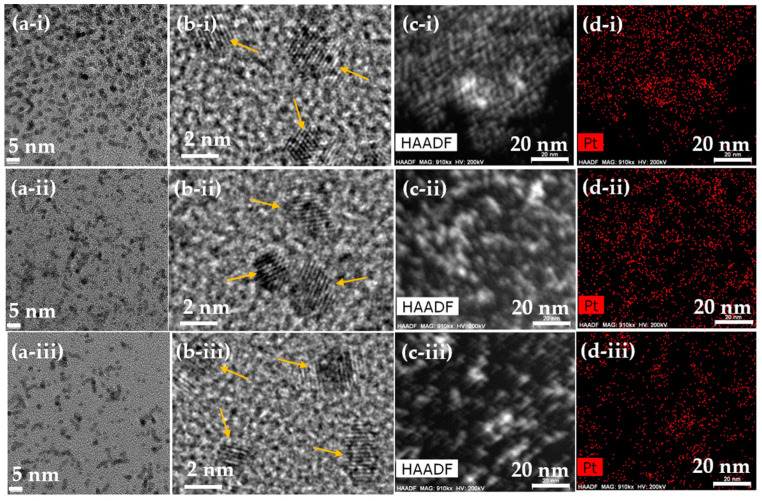

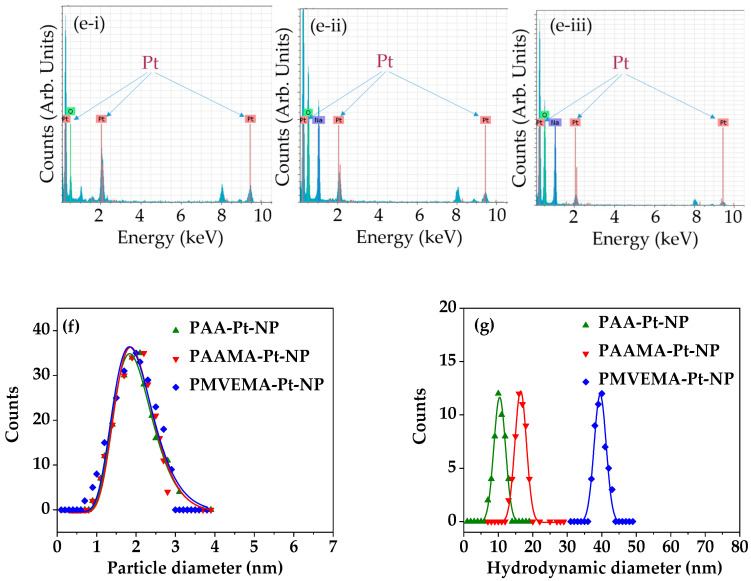
(**a**,**b**) HRTEM images, (**c**) HAADF-STEM images, (**d**) elemental mapping in HAADF-STEM images, and (**e**) EDS spectra for (i) PAA-, (ii) PAAMA-, and (iii) PMVEMA-coated Pt-NPs. Arrows in (**e**) indicate signals from Pt. (**f**) Particle diameter distributions and log-normal function fits to obtain d_avg_. (**g**) Hydrodynamic diameter distributions and log-normal function fits to obtain a_avg_.

The HRTEM images exhibited monodispersed ultrasmall particle size distributions for all polymer-coated Pt-NPs (polymer = PAA, PAAMA, and PMVEMA) (Figure 2a,b). In addition, the HAADF-STEM images (Figure 2c) confirmed monodispersed Pt-NPs in the NP samples. The Pt-elemental mapping over the HAADF-STEM images (Figure 2d) confirmed the presence of uniformly dispersed Pt-NPs in the NP samples. The EDS was also used to confirm the presence of Pt-NPs in the samples by observing transition peaks from Pt (Figure 2e). Fitting the log-normal function to the observed particle diameter distributions suggested that the average particle diameter (d_avg_) was approximately 2.0 nm for all polymer-coated Pt-NPs (Figure 2f and Table 1). Further, fitting the log-normal function to the observed hydrodynamic diameter distributions indicated that the average hydrodynamic diameters (a_avg_) were approximately 10.4, 20.5, and 37.5 nm for PAA-, PAAMA-, and PMVEMA-coated Pt-NPs, respectively (Figure 2g and Table 1), showing the proportionality of a_avg_ to the polymer size.

**Figure 3:**
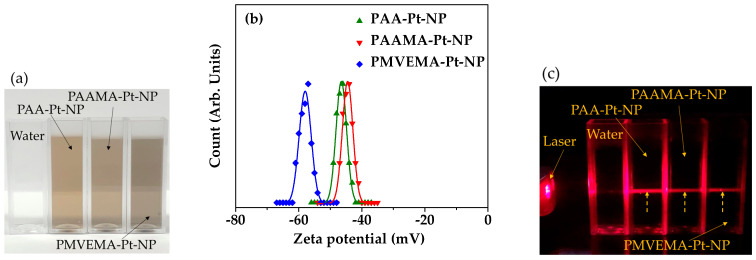
(**a**) Photographs of the diluted NP solution samples, showing no NP precipitation after synthesis for >1.5 years. (**b**) Zeta potential curves of the diluted NP solution samples and Gaussian function fits to obtain ζ_avg_. (**c**) Laser light scattering (indicated with dotted arrows) due to laser light collision with NP colloids in aqueous media, showing good colloidal dispersions, whereas no laser light scattering was observed for the vial containing triple-distilled water.

The photographs of NP sample solutions in Figure 3a indicate the lack of NP precipitation after preparing the polymer-coated Pt-NPs in aqueous media (>1.5 years). This highlights their excellent colloidal stability in aqueous media. This finding was also supported by highly negative zeta potentials (−46.5, −44.2, and −57.3 mV for PAA-, PAAMA-, and PMVEMA-coated Pt-NPs, respectively; Figure 3b and Table 1). Colloidal dispersion in aqueous media was confirmed by visible laser light scattering (called the Tyndall effect), i.e., laser light scattering was observed only for the vials containing polymer-coated Pt-NP colloidal samples, but not for the vial containing triple-distilled water (Figure 3c).

**Figure 4:**
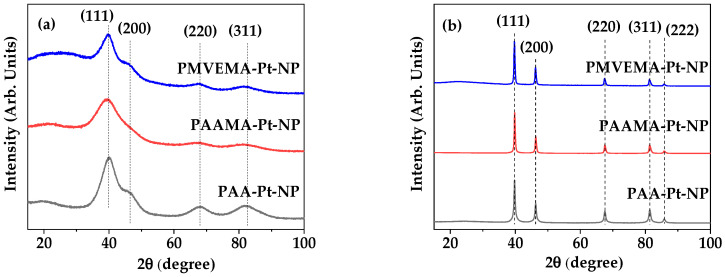
XRD patterns of the powder samples of PAA-, PAAMA-, and PMVEMA-coated Pt-NPs (**a**) before and (**b**) after TGA, up to 700 °C under nitrogen flow. All peaks could be assigned with (*hkl*) Miller indices of the fcc structure of bulk Pt metal.

The XRD patterns of all as-prepared powder samples were broad (Figure 4a) owing to ultrasmall particle sizes, as observed in the HRTEM images; all peaks could be assigned with (hkl) Miller indices, i.e., (111), (200), (220), and (311) of the face-centered cubic (fcc) structure of the bulk Pt metal, as labelled on the top of the peaks. Following TGA, all peaks became sharper owing to heat treatment up to 700 °C under nitrogen flow, and in addition, the small (222) peak also appeared (Figure 4b). This is due to crystal growth during heat treatment [46]. The estimated lattice constant (ℓ) of 3.911 Å was consistent with the reported value of 3.912 Å [47]. Using the formula of atomic radius r = √2ℓ/4 for the fcc structure, r was estimated to be 1.383 Å, which is consistent with the reported value of 1.385 Å [48].

### 3.2. Polymer-Coating Amount and Structure

**Figure 5:**
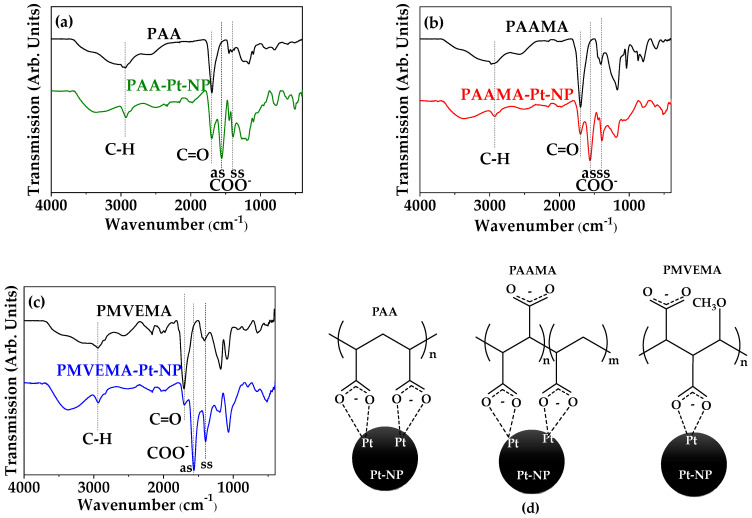
FT-IR absorption spectra of (**a**) free PAA and PAA-coated Pt-NPs, (**b**) free PAAMA and PAAMA-coated Pt-NPs, and (**c**) free PMVEMA and PMVEMA-coated Pt-NPs: “as” and “ss” indicate asymmetric and symmetric stretchings of COO^−^, respectively. (**d**) Schematic diagrams showing PAA-, PAAMA-, and PMVEMA-coating structures on Pt-NP surfaces: multiple bonds may exist between many COO^−^ groups of a polymer and many Pt atoms on the Pt-NP surface.

The polymer coating (polymer = PAA, PAAMA, and PMVEMA) on Pt-NP surfaces was investigated by recording the FT-IR absorption spectra and TGA curves. The characteristic vibrational absorption frequencies of the polymers were observed at ~2930 (C–H symmetric stretch), ~1695 (C=O symmetric stretch), ~1555 (COO^−^ antisymmetric stretch), and ~1390 cm^−1^ (COO^−^ symmetric stretch) in the FT-IR absorption spectra of polymer-coated Pt-NP samples, confirming successful polymer coating on the Pt-NP surfaces (Figure 5a–c). The observed absorption frequencies are listed in Table 2. The C=O symmetric stretch at ~1695 cm^−1^ was reduced in intensity and was also red-shifted and split into symmetric and antisymmetric stretches of COO^−^ after coating; these could be attributed to the electrostatic bonding between COO^−^ groups of the polymers and Pt atoms on the NP surfaces [49,50]. This corresponds to a hard acid (Pt atoms on the NP surfaces) hard base (COO^−^ groups of the polymers) type of bonding [51], as depicted in Figure 5d. Owing to the numerous COO^−^ groups per polymer, multiple bonds can exist between many COO^−^ groups of a polymer and many Pt atoms on a Pt-NP surface.

**Figure 6:**
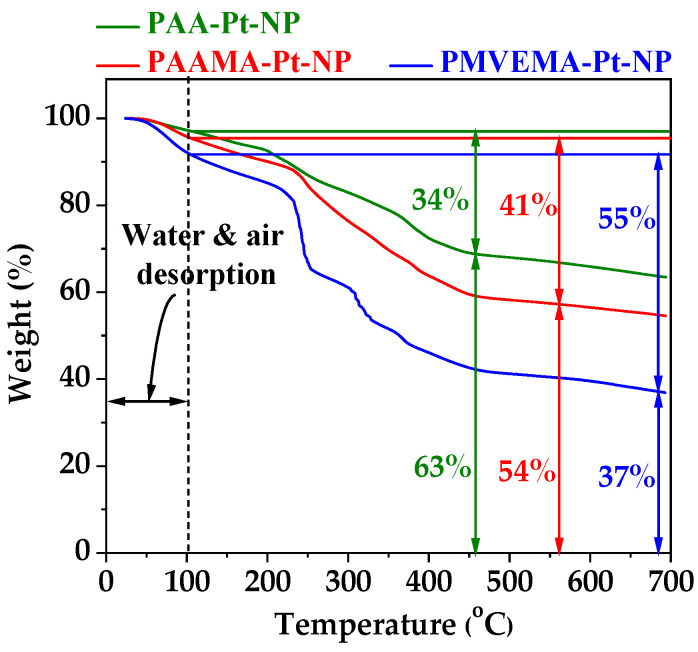
TGA curves of polymer-coated Pt-NPs (polymer = PAA, PAAMA, and PMVEMA).

The amount (P) of polymer coating on the Pt-NP surfaces in wt. %, ranging from 34 to 55%, was obtained from TGA curves by measuring mass losses after considering initial mass drops between room temperature and ~100 °C owing to water and air desorption from powder samples (Figure 6 and Table 3). The remaining mass drops were attributed to the removal of polymers from the Pt-NPs during heating to 700 °C under nitrogen flow. Grafting densities (σ), corresponding to the average number of polymers coating a NP unit surface area [52,53], were estimated to be 1.2, 1.0, and 0.1 nm^−^^2^ for PAA-, PAAMA-, and PMVEMA-coated Pt-NPs, respectively, using the Pt bulk density (21.45 g/cm^3^) [54], the d_avg_ values estimated from HRTEM imaging, and the *p* values obtained from the TGA curves. The average number (N_polymer_) of polymers coating an NP surface was estimated from the product, σ × NP surface area, in which the NP surface area was approximately estimated from πd_avg_^2^. The coating results are summarized in Table 3. Under these coating conditions, excellent colloidal stability was observed in aqueous media, indicating sufficient coating.

### 3.3. In Vitro Cellular Cytotoxicity Results

**Figure 7:**
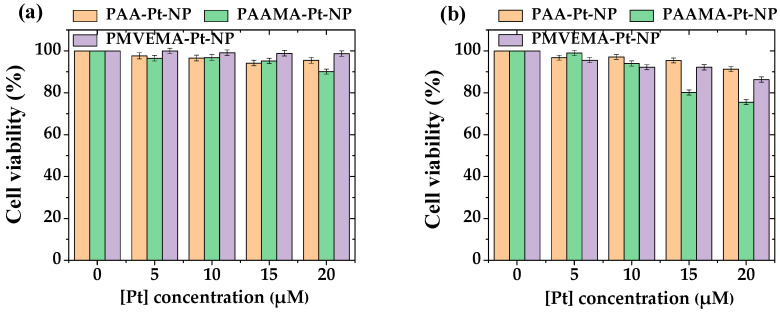
In vitro cellular cytotoxicity of PAA-, PAAMA-, and PMVEMA-coated Pt-NPs in (**a**) HepG2 and (**b**) HEK293 cells as a function of [Pt] concentration, showing low toxicity up to 20 μM [Pt].

The cellular cytotoxicity of polymer-coated ultrasmall Pt-NP samples was assessed by measuring the viability of HepG2 and HEK293 cells 48 h after incubation with the NP samples. As shown in Figure 7, cell viability was high (>75%) up to the tested concentration range (20 μM [Pt]), confirming the low toxicity of all polymer-coated ultrasmall Pt-NPs.

### 3.4. X-ray Phantom Images and X-ray Attenuation Power

**Figure 8:**
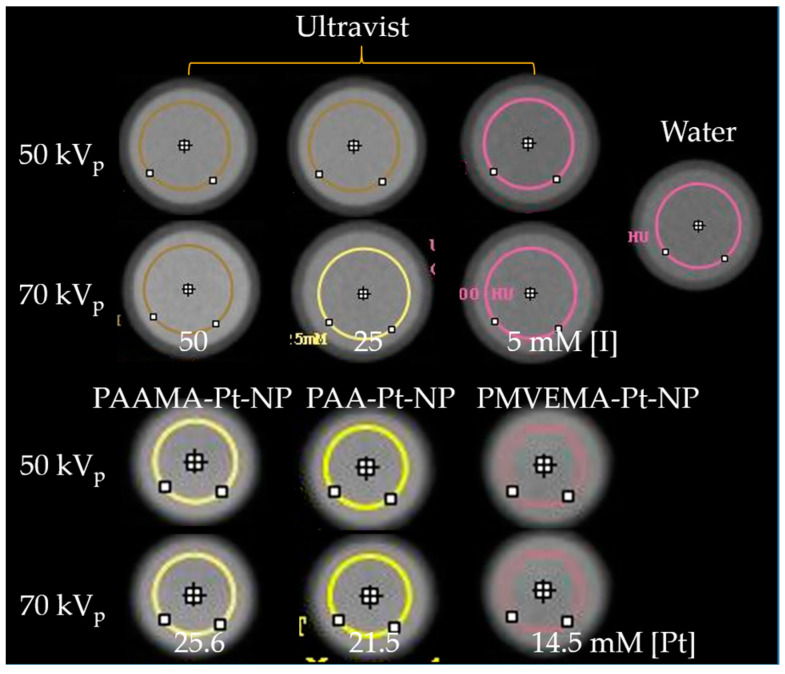
X-ray phantom images of polymer-coated Pt-NPs in aqueous media and Ultravist (X-ray source voltages, 50 and 70 kV_p_).

**Figure 9:**
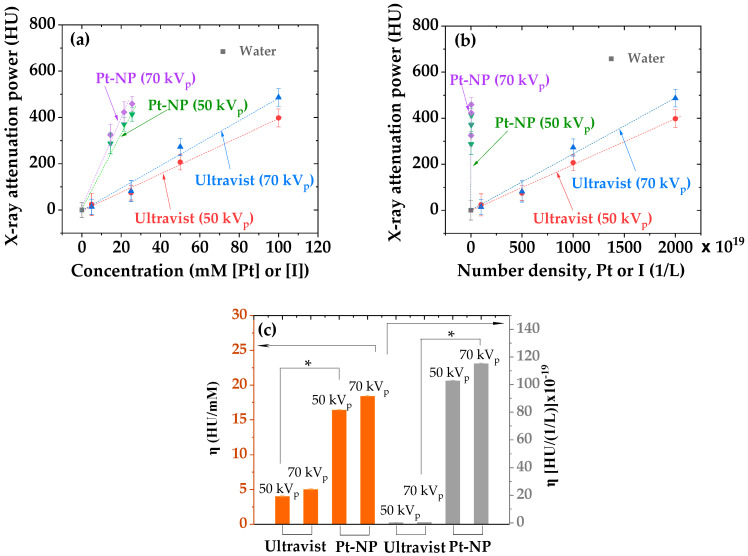
Plot of X-ray attenuation power as a function of (**a**) atomic concentration and (**b**) number density: the dotted lines indicate slopes [i.e., X-ray attenuation efficiency (η)]. (**c**) Plot of η of polymer-coated Pt-NPs and Ultravist in HU/mM (left) and HU/(1/L) (right) (X-ray source voltages, 50 and 70 kV_p_): (*: *p* * < 0.05, the significance of the results was confirmed using a *t*-test).

The X-ray attenuation properties of PAA-, PAAMA-, and PMVEMA-coated Pt-NPs in aqueous media were characterized by capturing X-ray phantom images at 50 and 70 kV_p_ (Figure 8). The results were compared with those of a commercial iodine contrast agent, Ultravist. The X-ray phantom images of the sample solutions were slightly brighter than those of Ultravist at a similar atomic concentration (Figure 8) because of the stronger X-ray attenuation coefficient of Pt compared with that of iodine [17]. In addition, the phantom images became slightly brighter with increasing X-ray source voltages from 50 to 70 kV_p_; this is owing to a wider X-ray energy range coverage at 70 kV_p_ and under such conditions, the X-ray attenuation coefficient becomes enhanced, thus providing brighter X-ray phantom images. To quantitatively investigate X-ray attenuation properties, X-ray attenuation power was estimated from the X-ray phantom images. As shown in Figure 9a, the X-ray attenuation power of the sample solutions was stronger than that of the Ultravist at the same atomic concentration. In addition, the X-ray attenuation power of the sample solutions and Ultravist was plotted as a function of the number density to further confirm the superiority of polymer-coated Pt-NPs to Ultravist (Figure 9b). To plot the X-ray attenuation power as a function of number density, the number density was estimated as the molar atomic concentration × 6.02 × 10^23^ ÷ N_atom_, where N_atom_ is the number of atoms per molecule or NP, which is three for Ultravist and ~(d_avg_/2r)^3^ [=(2.0/0.2766)^3^ = 378] for Pt-NPs (Table 4), in which 2r is the Pt atomic diameter of 0.2766 nm estimated from the lattice constant, as described before, and d_avg_ is the average NP diameter of 2.0 nm (Table 1) obtained from HRTEM imaging. As plotted in Figure 9b, the X-ray attenuation power of the sample solutions was significantly higher than that of Ultravist at the same number density. 

The X-ray attenuation efficiency (η), which corresponds to the molar X-ray attenuation power or X-ray attenuation power per number density, is a primary parameter for comparing the X-ray attenuation power of the materials. As a function of atomic concentration (slopes in Figure 9a), the η values of the Ultravist and sample solutions were estimated to be 4.0 and 16.4 HU/mM at 50 kV_p_, respectively, and 5.0 and 18.4 HU/mM at 70 kV_p_, respectively (Table 4). As a function of number density (slopes in Figure 9b), they were estimated to be 0.20 × 10^−20^ and 102.7 × 10^−19^ HU/(1/L) at 50 kV_p_, respectively, and 0.25 × 10^−20^ and 115.1 × 10^−19^ HU/(1/L) at 70 kV_p_, respectively (Table 4). Thus, the Pt-NP samples exhibited approximately four times and 500 times higher X-ray attenuation power than Ultravist at the same atomic concentration and number density, respectively. The η values are plotted in Figure 9c, clearly showing the higher η values of the Pt-NP samples. These results confirm the superiority of polymer-coated Pt-NPs to iodine contrast agents.

## 4. Discussion

PAA-, PAAMA-, and PMVEMA-coated ultrasmall Pt-NPs were synthesized using a simple one-pot polyol method. This method is considerably simpler compared with previously reported methods [16,32,33,34,35,36]. All polymer-coated Pt-NPs were stable in colloidal form in aqueous media and nearly non-toxic. As displayed in Figure 1, all of the polymers used contain numerous hydrophilic –COOH groups; consequently, they can strongly bind to the Pt-NPs and attract numerous water molecules around the NPs, thereby providing large hydrodynamic diameters and excellent colloidal stability. In addition, the hydrodynamic diameter increased with increasing polymer size from PAA → PAAMA → PMVEMA. This is because larger polymers are bulkier and contain more hydrophilic -COOH groups that can attract water molecules around the Pt-NPs, thus providing larger hydrodynamic diameters.

The stronger X-ray attenuation power of the sample solutions (compared to that of Ultravist) was indicated by the brighter phantom images of the sample solutions at similar atomic concentration (Figure 8). We quantitatively validated this by estimating the X-ray attenuation power values from the phantom images and plotting them as a function of atomic concentration [Pt] or [I] (Figure 9a and Table 4). Furthermore, the X-ray attenuation power of the sample solutions was significantly higher than that of Ultravist at the same number density (Figure 9b). This result is important because the osmolality and viscosity of the contrast agents mainly depend on the number density rather than the atomic concentration [23]. Contrast agents with high osmolality and viscosity can cause side effects in patients with kidney injury [55]. However, NP contrast agents can have lower viscosity and osmolality than iodine contrast agents owing to their lower number density at the same atomic concentration [23]. In addition, the injection dose of Pt-NP contrast agents can be further reduced because of the higher X-ray attenuation coefficient of Pt compared with that of iodine [17].

The X-ray attenuation efficiency (η) is a primary parameter indicating the performance of X-ray contrast agents. Our results were compared with those of other studies [16,32,33,34,35,36]. As shown in Table 5, our Pt-NP η value was higher compared with other values at the same X-ray source voltage (kV_p_) because the η value increases with X-ray source voltage. This higher value can be explained as follows. The η value decreases with particle size but increases with colloidal stability. For NPs with poor colloidal stability, the precipitated NPs poorly contribute to X-ray attenuation, resulting in low η values. Our hydrophilic polymer-coated ultrasmall Pt-NPs exhibited excellent colloidal stability (i.e., no precipitation after synthesis), thus rendering a high η value. The internal Pt atoms in larger NPs contribute less to the X-ray attenuation compared with surface Pt atoms because of the penetration depth limit of X-rays in metals. As shown in Table 5, our Pt-NPs have smaller particle diameters compared with those of other Pt-NPs, explaining the obtained higher η value compared with others. Thus, given all of the above findings, the hydrophilic polymer-coated ultrasmall Pt-NPs (polymer = PAA, PAAMA, and PMVEMA) synthesized in this study constitute promising potential high-performance X-ray contrast agents.

## 5. Conclusions

In summary, we used for the first time a one-pot polyol method to synthesize ultrasmall Pt-NPs coated with three types of hydrophilic and biocompatible polymers (i.e., PAA, PAAMA, and PMVEMA). We evaluated their cytotoxicity using HepG2 and HEK293 cell lines, and characterized their physicochemical and X-ray attenuation properties.

1. The observed average particle diameter was nearly monodispersed and ultrasmall (i.e., 2.0 nm) for all polymer-coated Pt-NPs;
2. Highly negative zeta potentials (<−40 mV) were observed for all polymer-coated Pt-NP solution samples owing to the coating of hydrophilic and biocompatible polymers on the NP surfaces. This led to excellent colloidal stability (no precipitation after synthesis for >1.5 years). Furthermore, all polymer-coated Pt-NP solution samples exhibited low toxicity (>75% cell survival) up to the tested concentration range of 20 μM [Pt], indicating their suitability for biomedical applications;
3. The X-ray attenuation power of all polymer-coated Pt-NP solution samples was ~4 times higher than that of the commercial iodine contrast agent Ultravist at the same atomic concentration and ~500 times higher at the same number density, confirming the superiority of the polymer-coated Pt-NPs to iodine contrast agents and thus, their potential as viable high-performance CT contrast agents.

## Figures and Tables

**Table 1:** Average particle diameters (d_avg_), average hydrodynamic diameters (a_avg_), and zeta potentials (ζ_avg_) of polymer-coated Pt-NPs.

Coating Polymer	d_avg_ (nm)	a_avg_ (nm)	ξ_avg_ (mV)
PAA	2.0 ± 0.2	10.4 ± 1.0	−46.5 ± 1.0
PAAMA	2.0 ± 0.2	20.5 ± 1.0	−44.2 ± 1.0
PMVEMA	2.0 ± 0.2	37.5 ± 1.0	−57.3 ± 1.0

**Table 2:** Observed FT-IR absorption frequencies in cm^−1^.

	PAA	PAAMA	PMVEMA	PAA-Pt-NPs	PAAMA-Pt-NPs	PMVEMA-Pt-NPs
C–H stretch	2940 ± 5	2935 ± 5	2942 ± 5	2926 ± 5	2922 ± 5	2935 ± 5
C=O stretch	1697 ± 5	1693 ± 5	1699 ± 5	1697 ± 5	1693 ± 5	1699 ± 5
COO^−^ antisymmetric stretch	-	-	-	1553 ± 5	1556 ± 5	1560 ± 5
COO^−^ symmetric stretch	-	-	-	1398 ± 5	1385 ± 5	1390 ± 5

**Table 3:** Summary of the surface-coating amount.

Coating Polymer	Surface-Coating Amount		
	P ^1^ (wt. %)	σ ^2^ (nm^−2^)	N_polymer_^3^
PAA	34 ± 1	1.2 ± 0.1	12.4 ± 0.1
PAAMA	41 ± 1	1.0 ± 0.1	11.0 ± 0.1
PMVEMA	55 ± 1	0.10 ± 0.05	1.1 ± 0.1

^1^ Average surface-coating amount per NP in wt. %. ^2^ Average number of polymers coating an NP unit surface area. ^3^ Average number of polymers coating an NP.

**Table 4:** Summary of the X-ray attenuation properties at 50 and 70 kV_p_.

Chemical	N_atom_	Concentration (mM [Pt] or [I])	Number Density (1/L)	X-ray Attenuation Power (HU)		X-ray Attenuation Efficiency (η)			
				50 kV_p_	70 kV_p_	(HU/mM)		[HU/(1/L)] × 10^−19^	
						50 kV_p_	70 kV_p_	50 kV_p_	70 kV_p_
PAA-Pt-NP	378 ± 5	21.5 ± 0.5	3.4 ± 0.1 × 10^19^	371 ± 30	423 ± 45	16.4 ± 0.1	18.4 ± 0.1	102.7 ± 0.5	115.1 ± 0.5
PAAMA-Pt-NP	378 ± 5	25.6 ± 0.5	4.1 ± 0.1 × 10^19^	413 ± 30	459 ± 32				
PMVEMA-Pt-NP	378 ± 5	14.5 ± 0.5	2.3 ± 0.1 × 10^19^	288 ± 45	325 ± 46				
Ultravist	3	100.0 ± 0.5	20.0 ± 0.1 × 10^21^	398 ± 39	487 ± 38	4.0 ± 0.1	5.0 ± 0.1	0.20 ± 0.01	0.25 ± 0.01
	3	50.0 ± 0.5	10.0 ± 0.1 × 10^21^	207 ± 34	273 ± 37				
	3	25.0 ± 0.5	5.0 ± 0.1 × 10^21^	75 ± 32	82 ± 46				
	3	5.0 ± 0.5	1.0 ± 0.1 × 10^21^	24 ± 48	14 ± 33				
Water	-	-	-	0 ± 32	0 ± 42	-	-	-	-

**Table 5:** Comparison of X-ray attenuation efficiency (η) with other studies.

Pt-NP Type	Coating Ligand	d_avg_ (nm)	η (Hu/mM)	Ref.
Mesoporous Pt-NP	Ascorbic acid	70	3.0 at 120 kV_p_	[16]
Spherical Pt-NP	Bovine serum albumin	2.1	16.8 at 120 kV_p_	[32]
Spherical Pt-NP	Extract from Prosopis farcta fruits	3.8	6.9 at 80 kV_p_	[33]
Mesoporous Pt-NP	Polyethylene glycol	94	5.5 at 120 kV_p_	[34]
Spherical Pt-NP embedded in ~50 nm mesoporous silica NP	Polyethylene glycol	3	3.0 at 70 kV_p_	[35]
Pt nanoworm	Polyethylene glycol	~3 × ~10	4.7	[36]
Spherical Pt-NP	PAA, PAAMA, PMVEMA	2.0	16.4 at 50 kV_p_, 18.4 at 70 kV_p_	This study

## Data Availability

The data presented in this study are available on request from the corresponding authors.
